# Can social network analysis help to include marginalised young women in structural support programmes in Botswana? A mixed methods study

**DOI:** 10.1186/s12939-019-0911-8

**Published:** 2019-01-18

**Authors:** David Loutfi, Neil Andersson, Susan Law, Jon Salsberg, Jeannie Haggerty, Leagajang Kgakole, Anne Cockcroft

**Affiliations:** 1 Department of Family Medicine, McGill University, 5858 chemin de la Côte-des-Neiges, 3rd floor, Montreal, Quebec H3S 1Z1 Canada; 2 Centro de Investigación de Enfermedades Tropicales (CIET), Universidad Autónoma de Guerrero, Acapulco, Mexico; 3 Trillium Health Partners, Institute for better health, 100 Queensway West, 6th Floor, Mississauga, ON L5B 1B8 Canada; 4 Institute for Health Policy, Management and Evaluation, University of Toronto, 4th Floor, 155 College Street, Toronto, ON M5T 3M6 Canada; 5 Graduate Entry Medical School, University of Limerick, Plassey Park, Co. Limerick, V94 T9PX Ireland; 6 CIET Trust Botswana, PO Box 1240, Gaborone, Botswana

**Keywords:** HIV, Prevention, Social networks, Recruitment, Young women, Marginalised populations

## Abstract

**Background:**

In Botswana, one fifth of the adult population is infected with HIV, with young women most at risk. Structural factors such as poverty, poor education, strong gender inequalities and gender violence render many young women unable to act on choices to protect themselves from HIV. A national trial is testing an intervention to assist young women to access government programs for returning to education, and improving livelihoods. Accessing marginalised young women (aged 16–29 and not in education, employment or training) through door-to-door recruitment has proved inefficient. We investigated social networks of young women to see if an approach based on an understanding of these networks could help with recruitment.

**Methods:**

This mixed methods study used social network analysis to identify key young women in four communities (using in-degree centrality), and to describe the types of people that marginalised young women (*n* = 307) turn to for support (using descriptive statistics and then generalized linear mixed models to examine the support networks of sub-groups of participants). In discussion groups (*n* = 46 participants), the same young women helped explain results from the network analysis. We also tracked the recruitment method for each participant (door to door, peers, or key community informants).

**Results:**

Although we were not able to identify characteristics of the most central young women in networks, we found that marginalised young women went most often to other women, usually in the same community, and with children, especially if they had children themselves. Rural women were better connected with each other than women in urban areas, though there were isolated young women in all communities. Peer recruitment contributed most in rural areas; door-to-door recruitment contributed most in urban areas.

**Conclusions:**

Since marginalised young women seek support from others like themselves, outreach programs could use networks of women to identify and engage those who most need help from government structural support programs. Methods that rely on social networks alone may be insufficient, and so a combination of approaches, including, for instance, peers, door-to-door recruitment, and key community informants, should be explored as a strategy for reaching marginalised young women for supportive interventions.

**Electronic supplementary material:**

The online version of this article (10.1186/s12939-019-0911-8) contains supplementary material, which is available to authorized users.

## Background

The HIV epidemic is far from over, and in southern Africa the continuing rate of new infections among young people, especially young women, is a serious concern [1]. Prevention efforts need to include structural, as well as biomedical and behavioural, interventions [2].

In Botswana, one fifth of the adult population is infected with HIV, with young women (15–30) most at risk [3]. Structural factors such as poverty, poor education, strong gender inequalities and gender violence render many young women unable to act on choices to protect themselves from HIV [4, 5]. The Inter-ministerial National Structural Intervention Trial (INSTRUCT) addresses this choice disability and the associated HIV risk among young women (ISRCTN 54878784). The complex intervention recruits marginalised young women to workshops that put them in touch with government structural support programmes and helps them to benefit from these programmes. The intervention also builds an enabling environment for young women to exercise protective choices, promoting evidence-based discussions across the community about HIV, transactional sex, and gender violence. The intervention works in tandem with government departments to make their support programmes more accessible to young women.

Existing government structural support programmes include help to improve educational qualifications, to start small enterprises, and to gain employment skills through apprenticeships. The government programmes, however, were not designed to reduce HIV risk or to benefit young women in particular, and marginalised young women (defined here as aged 16–29 years, not in paid work and not in education) rarely access these support programmes [6]. These young women are at high risk of contracting HIV and have little support that could enable them to rely less on transactional sex. INSTRUCT aims to help these young women to access the programs to reduce their reliance on transactional sex and prevent HIV. Key to success is identifying and engaging the most vulnerable young women.

Our study builds on research using social networks to identify and to recruit hard-to-reach populations, such as injecting drug users [7] and men who have sex with men (MSM) [8–12]. Even a study of jazz musicians, a more unusual hard-to-reach population, has used social networks as a method to recruit a more full population [13]. Studies of MSM found that identifying central men in a network and training them about HIV prevention improved safe sex practices in intervention networks more than in control networks [9, 12]. An initial pilot study in four communities in one district of Botswana demonstrated the feasibility of recruiting young women for a survey about their social networks (Loutfi D, Andersson N, Law S, Kgakole L, Salsberg J, Haggerty J, Cockcroft A: Reaching marginalised young women for HIV prevention in Botswana: a pilot social network analysis, forthcoming). Lessons learned from the pilot study included the time needed to recruit participants in a community, and the need to engage key community members to help recruit potential participants. The pilot provided some preliminary indications about the support networks of marginalised young women.

The objective of this mixed methods study was to investigate and understand the social networks of marginalised young women in rural and urban communities in Botswana, in the expectation that this may indicate ways to reach the most marginalised among them and include them into government support programmes – including through the INSTRUCT intervention – that could help to reduce their choice disability and HIV risk.

## Methods

### Recruitment and data collection for survey of young women

We trained young women from one district in Botswana to undertake a survey of the social networks of marginalised young women in two rural and two urban communities in the district, with populations ranging from a few hundred to a few thousand. The field team used three methods to try to identify all the eligible young women (aged 16–29, not in paid work and not in education). These eligibility criteria selected young women who could most benefit from the government support programs – given their lack of employment and limited education. We did not attempt to obtain a representative sample; rather, we attempted to reach every eligible young woman in each community. The field team asked key informants, such as the village chief, village development committee members, social workers, and health education assistants from the local clinic, about eligible young women. They enquired door to door. And they asked survey respondents to identify other young women like themselves living in the community. The team returned over 4–5 days to look for identified eligible young women, and in some cases tried to contact them by phone to arrange an interview.

The 2 urban locations were neighbourhoods in a town of about 20,000 people. Urban 1 is more affluent than Urban 2, and although both have access to the same health and other government services, Urban 1 is more central than Urban 2 that is more on the periphery of the town. Rural 1 is a medium sized village of a few thousand residents; Rural 2 has a few hundred. Rural 1 is accessible by paved roads, and there is regular transport (mini-buses) to and from the village. Rural 2 is only accessible by a dirt road, and transport to and from the village is much more difficult for residents. It is about an hour’s drive from these villages to a larger urban center. All communities have clinics and schools and some other government services, but the main offices to apply to many support programmes are only in the urban locations.

The survey questionnaire collected demographic information about the respondents; it asked them to identify who they turned to for information about employment or educational opportunities, who they socialized with, and who they turned to for emotional support; and it asked them for demographic information about the people they turned to for support. We asked about three types of support. *Informational support:* Sometimes people ask other people for information or advice about important decisions in their life, for example education or employment opportunities. Who do you usually ask for advice in these types of situations? *Socialising:* Who do you usually socialize with? *Emotional support:* Sometimes, people discuss important personal matters with other people such as problems with boyfriends, friends, or with family. Who do you discuss such personal matters with? The interviewers recorded how they identified each respondent (door-to-door recruitment, peer recruitment, or from key community informants). When participants identified young women in their support networks, aged 16–29, that lived in their community, the tablet automatically prompted the interviewer to ask how best to contact this person for a potential interview. The young women participants gave oral informed consent for the interview in Setswana, which took about 1 hour.

The electronic data collection used the Open Data Kit [14, 15]. Interviewers recorded responses on hand-held android tablets and supervisors checked completed records before sending them to a server in Gaborone, from where we downloaded the dataset for analysis.

### Discussion groups

We presented and discussed a summary of key findings with groups of survey participants in each of the four communities to seek feedback and enable a deeper understanding of the findings. The group facilitator, a young woman from the field team, knew many of the young women and had helped some of them to apply to government support programmes. She invited participants to the discussions in-person and by telephone. The hour-long discussions in Setswana took place either in the Kgotla, a place in each community where public meetings are held, or in a government office nearby. We aimed for groups of 6–8 participants as in focus group research [16]. We provided transport to and from the meeting and a meal for participants.

**Fig. 1:**
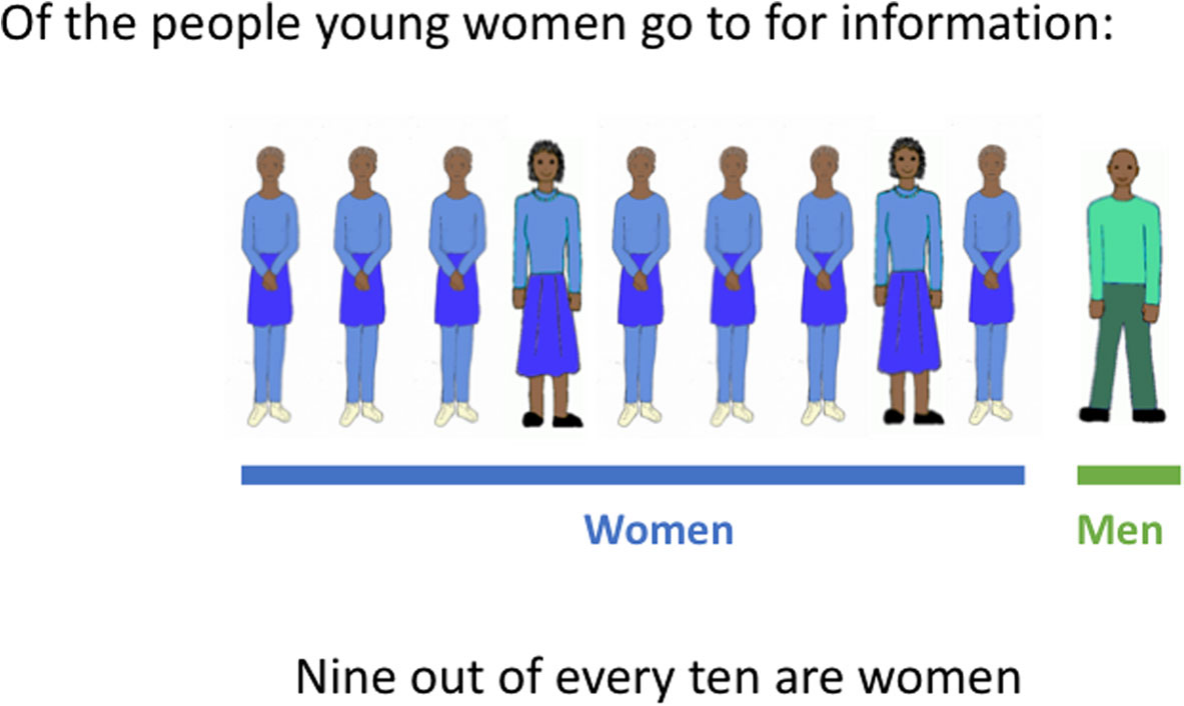
Example of a graphic display of findings from the social network analysis used in the discussion groups

The facilitator presented each key finding in turn, using large engaging graphics (Fig. 1 is one example of the graphics we used) displayed on a flip-chart stand. Additional file 1 lists the statements the facilitator made about the survey respondents and the sort of people in their support networks. The facilitator asked, about each finding: “What do you think about this finding, based on your experience and what you know about your community?” Participants discussed each finding in turn, exploring whether they agreed with the findings or not and why. The facilitator then asked: “Why do you think young women choose people like themselves or other female family members to go to for information?” The discussion guide for the facilitator included prompts for each of the questions to tease out views from the participants. Two note takers captured the content of the discussions and recorded relevant quotes verbatim. The facilitator, note takers, and a researcher met after each discussion group to finalise the report and produce an English translation.

### Analysis

The analysis had three parts. First we examined networks of young women to identify the most central participants, and define their characteristics. Second, we examined the broader networks of the young women, and the characteristics of the people that they turned to for support. Third, we used qualitative findings from the discussion groups to give context and help in interpretation of the quantitative findings.

### Sociometric analysis

We created network graphs and used sociometric analysis to explore how the marginalised young women were connected *to each other*. Sociometric analysis examines the structure of a network graph to describe the relationships between participants [17]. In our network graphs, nodes represent individual participants, and edges (or lines) indicate contact between individuals seeking support.

We attempted to identify people in the network that could spread information to the rest of the network. To determine who these might be, we calculated in-degree centrality for each. This counts the number of times each participant is listed by others in the network as a source of support [18]. We used linear regression to examine characteristics of the participants linked to their individual centrality. For example, we asked if the most educated participants were also the most central. The characteristics of participants (independent variables) included: age of the participants (< 21 or > =21), extreme poverty (had enough food to eat in the last week or not), having children vs. not having children, education (completed secondary education or not), and cohabiting with a partner or not. We did not collect data on ethnicity or religion because this area of Botswana is almost exclusively Christian and Batswana (majority ethnic group in Botswana). The dependent variable was in-degree centrality.

To examine the overall structure of the network, we used graph *centralization*, a concept that describes the whole network graph, rather than in-degree *centrality* which measures how central each individual participant is. Graph centralization ranges from 0 to 1 and measures whether centrality is concentrated in a few individuals (closer to 1) or spread out evenly between individuals (closer to 0) [18].

### Ego-centric analysis

We explored the broader support networks of marginalised young women, using descriptive statistics to describe who they go to for support, not limited to other young women. Bivariate and multivariate (mixed effects logistic regression) analyses explored whether young women go to different kinds of people for support depending on their own personal characteristics and the type of community. For example, we examined the factors related to young women going to women or men for support. The dependent variables (characteristics of support person) included: gender (male or female), age (within 5 years of participant or not), relationship type (friend or relative), living in the same community or not (community refers to villages in rural areas, and neighbourhoods in urban areas), having children or not, and having completed secondary education or not. The independent variables (characteristics of the participants or community) were location (urban/rural), age (< 21 or > =21), poverty (enough food in the last week or not), cohabiting with a partner or not, having children or not, and completed secondary education or not. We selected the independent variables through discussion with local partners based on their understanding of what factors in the local context might determine who young women might turn to for support.

For each of six outcomes, we started with a saturated model including all six independent variables. We used step-wise deletion of non-significant variables, at each step deleting the variable with the lowest value of chi-square, to reach final models including only variables significantly associated (at 5% level) with the outcome [19].

In this dataset, since each participant named multiple people for support, each participant and their characteristics are repeated several times. This repetition could lead to overestimating the confidence in some measures of association between our independent and dependent variables. We managed this using generalized linear mixed models, in which each individual is treated as a cluster [20]. In a separate analysis, we included community as a cluster to account for community level factors such as location (urban/rural) that are the same for all participants in that community. More detail about the clustered analyses is given in Additional file 2.

We used R for analysis, including the igraph package for the sociometric analysis, and the lme4 package for the multivariate analysis [21–23].

### Qualitative analysis

We carried out a thematic analysis of the discussion group notes using pre-defined themes derived from the quantitative analysis [24]. Each theme referred to one of the findings that we presented during the discussion groups (e.g. male vs. female support, age of support person, etc.) We examined the range of perspectives in the qualitative data alongside the quantitative findings to provide context and nuance to the quantitative findings. We used NVivo for qualitative analysis [25].

## Results

### Recruitment of survey respondents

Field workers identified 344 eligible survey participants and interviewed 307 of these young women (89%). Only four refused; most of the other 33 were not in their community during the 4–5 days of data collection. Of those who participated, 46% (140) came from door-to-door visits, 41% (127) were identified by other respondents, and 13% (40) were identified by key community informants. In rural communities, 34% (67) of 196 respondents came from door-to-door visits, 52% (102) from other respondents, and 14% (27) from key community informants. In urban communities, of 111 respondents, 66% (73) came from door-to-door visits, 23% (26) from other respondents, and 11% (12) from key community informants.

For the discussion groups, we recruited 11 participants in community Urban 1 with a mean age (sd) of 25 (3.3); in community Urban 2 there were 6 participants with a mean age (sd) of 23 (2.8); in community Rural 1 there were 8 participants with a mean age (sd) of 23 (3.3); in community Rural 2 there were 21 participants with a mean age (sd) of 21 (2.3).

Table 1 shows characteristics of the survey respondents. These characteristics were similar across the four communities. About one-fifth were co-habiting and two-thirds had at least one child. Only one-third had completed secondary education.

**Table 1:** Characteristics of survey respondents in two urban and two rural sites

	Percent (number) of respondents				
	Total	Urban 1	Urban 2	Rural 1	Rural 2
	*n* = 307	*n* = 64	*n* = 47	*n* = 103	*n* = 93
Mean age (sd) [years]	22.7 (3.6)	22.5 (3.6)	22.4 (3.3)	23.4 (3.7)	22.2 (3.3)
*Marital status*					
Single	75.6 (232)	82.8 (53)	72.3 (34)	74.8 (77)	73.1 (68)
Cohabiting, not married	22.8 (70)	17.2 (11)	27.7 (13)	22.3 (23)	24.7 (23)
Married	0.7 (2)	0.0 (0)	0.0 (0)	1.0 (1)	1.1 (1)
Separated from partner	1.0 (3)	0.0 (0)	0.0 (0)	1.9 (2)	1.1 (1)
Have a regular partner	59.0 (181)	54.7 (35)	51.1 (24)	60.2 (62)	64.5 (60)
Partner > 5 years older *n* = 181	41.4 (106)	25.7 (26)	50.0 (12)	46.8 (33)	41.7 (35)
*Number of children*					
0	37.1 (114)	51.6 (33)	36.2 (17)	31.1 (32)	34.4 (32)
1	32.9 (101)	28.1 (18)	36.2 (17)	35.0 (36)	32.3 (30)
2	22.8 (70)	17.2 (11)	19.1 (9)	26.2 (27)	24.7 (23)
3 or more	7.1 (22)	3.2 (2)	8.5 (4)	7.8 (8)	8.7 (8)
Low education (incomplete secondary or less)	65.1 (223)	62.2 (40)	53.2 (25)	64.1 (66)	74.2 (69)
Very poor (not enough food in last week)	15.7 (48)	18.8 (12)	19.1 (9)	12.6 (13)	15.2 (14)

### Sociometric analysis (examining centrality)

**Fig. 2:**
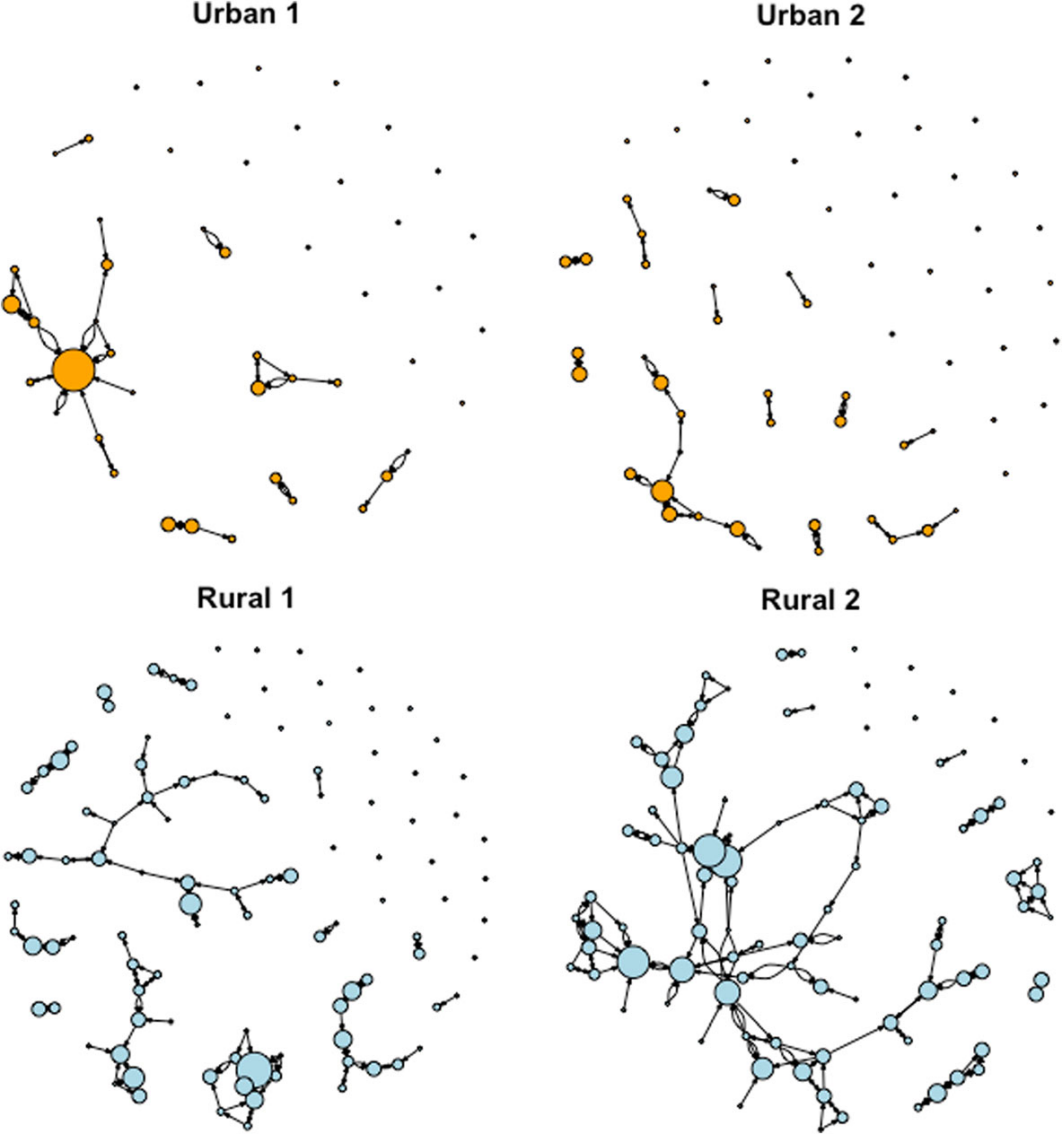
Support networks of marginalised young women in 2 urban and 2 rural communities. Legend: Each node represents a participant. Arrows indicate contact between individuals for social support. Node size indicates centrality (i.e. being sought for support)

Figure 2 shows the network graphs for each community. We combined the graphs for the information, socializing, and emotional support networks to show all connections between participants. The number of times participants were sought for support (in-degree centrality), is indicated by the size of the nodes in Fig. 2. Bivariate analysis indicated that the poorest participants were never highly central, though this was not significant in multivariate analysis. A linear regression did not identify any characteristics of individuals (age, poverty, presence of children, education, or cohabitation) that were significantly associated with centrality. In a specific community it would be useful to target individuals with high in-degree centrality to spread information, but we cannot predict from our analysis the characteristics of individuals likely to have high in-degree centrality.

The graphs show participants were better connected in rural than in urban areas and there were proportionally more isolated participants in urban areas (Fig. 2). Table 2 shows metrics derived from the graphs. The number of nodes (participants), and number of ties (support between participants) provides a basic description of the diagrams [26]. The proportion of isolates helps understand the proportion who are harder to reach. Graph centralization helps us to understand whether network ties are focused on one person who might be useful for spreading information or whether the ties are distributed amongst many people [18]. Community Urban 1 has a higher centralization score, and one node appears much larger than the others. Communities Rural 1, Rural 2, and Urban 2 have many nodes that are equally central; graph centralization is lower, because the centrality is more evenly spread out. This suggests that reaching out through one central person would be more effective only in community Urban 1; in the other communities there is not a central person who could share information more effectively than the rest.

**Table 2:** Network measures

	Urban 1	Urban 2	Rural 1	Rural 2
Number of nodes	47	64	103	93
Number of ties	46	47	140	180
Isolates as % of total nodes (n)^a^	38% (18)	45% (29)	26% (27)	10% (9)
Network centralization ^b^	0.22	0.07	0.08	0.07

^a^Isolates are nodes that have no connections to any others in the graph

^b^Closer to 0 indicates more evenly distributed node centrality. Closer to 1 indicates that centrality is concentrated in a few individuals

### Ego-centric analysis

As shown in Table 3, marginalised young women mostly went for support to other women, to people living in the same community, and to people with children. They went to others within 5 years of their own age about half the time. When the support persons were not within 5 years of their age, they were almost always older (96.3%). Young women went to people without a secondary education a bit more than half the time, and they went to relatives more often than to friends.

**Table 3:** Characteristics of the people young women go to for support (*n* = 1923)

Characteristic	Percent (number)
Female	86.7 (1668)
Has children	66.0 (1270)
Similar Age (+ − 5 years)	57.0 (1096)
Low education (incomplete secondary) (*n* = 1909)	57.5 (1097)
Live in the same community	66.8 (1285)
*Type of relationship (n = 1920)*	
Relative	58.3 (1119)
Friend and other	41.7 (801)
*Role in the community*	
Public/community^a^	3.3 (64)
Private sector employee	18.3 (351)
Government employee	6.0 (115)
Student/Volunteer	11.0 (212)
No specific role	61.4 (1181)

^a^Public/community includes pastors, teachers, social workers, health workers, traditional doctors, Kgosi (chief), and village development committee members

**Fig. 3:**
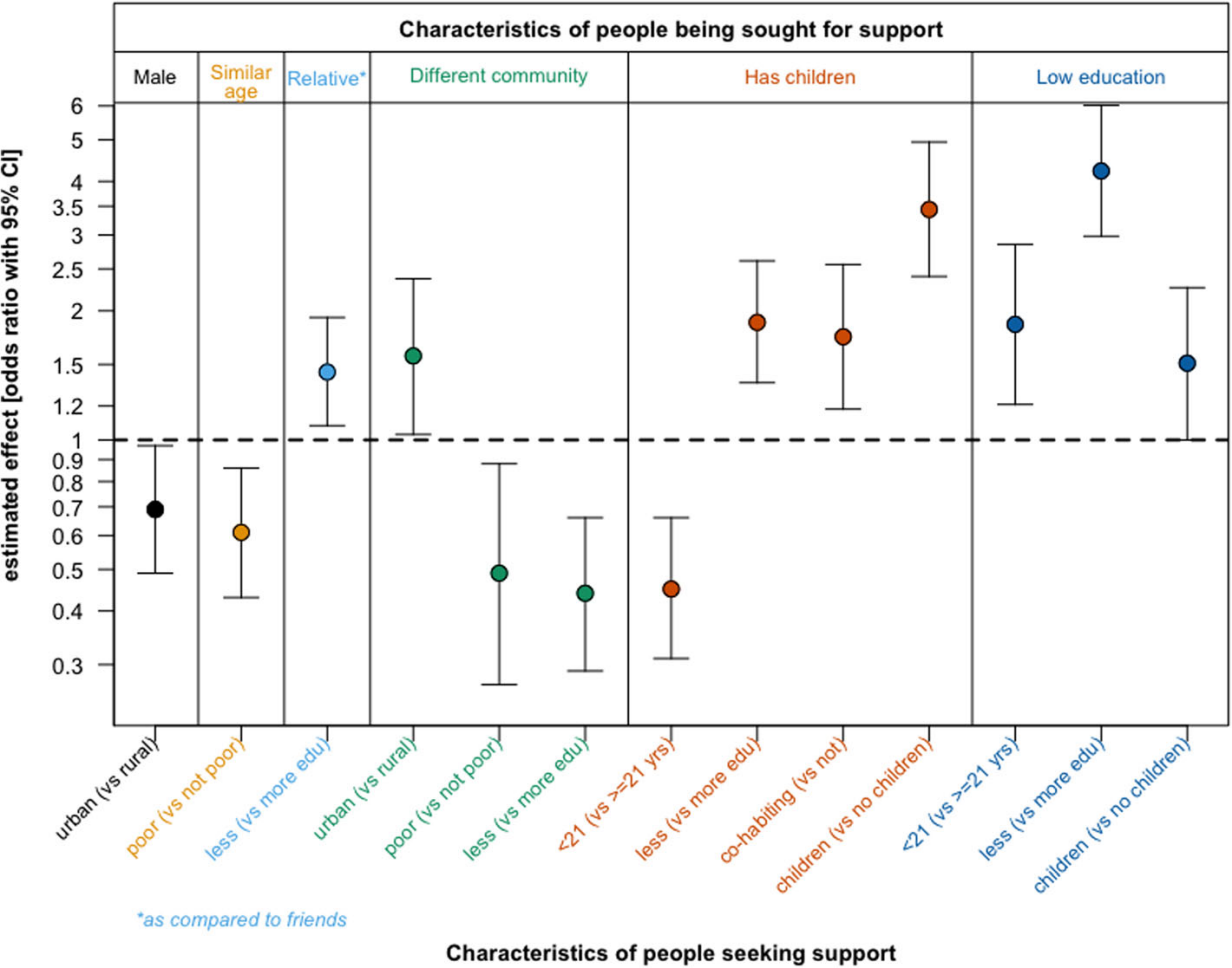
Multivariate models explaining who sub-groups of marginalised young women turn to for support. Legend: This chart shows the odds ratios and 95% confidence intervals for the variables that were significant in our final models. Along the top are the six dependent variables (one for each model). Along the bottom are the independent variables

Figure 3 shows the odds ratios and 95% confidence intervals for the variables in the final models of the logistic regression analyses for each of the six outcomes (characteristics of the support persons). The initial saturated models and the final models as tables are shown in Supplementary Table 1, Additional file 3. A sensitivity analysis, with age cut-offs for age categories of participants set at 19, 20, and 21, gave similar findings in the models.

Young women in urban areas were less likely to turn to men for support than were rural young women (OR = 0.69, 95% CI 0.49–0.97). The discussion groups were clear that young women get most of their support from other women. One young woman said: “We would rather run into a tree with thorns, than to men.”

The poorest participants were less likely to turn to someone within 5 years of their own age (OR = 0.61, 95% CI 0.43–0.86) (turning instead to someone older). Discussing these findings, young women indicated that, whether poor or not, many of them did not seek support from people that were older because they might get a critical reaction. “Elders are impatient with us.”

Participants that had not completed secondary education were more likely to go to a relative rather than a friend for support than those that had completed secondary education (OR = 1.44, 95% CI 1.08–1.93). The discussion around approaching friends or relatives was mixed. One participant said: “You can’t trust someone you’re not related to. Sometimes if you go to someone you’re not related to, they will dismiss you.” Another said, “I would not go to a relative because a relative can ridicule you if you succeed, saying you are there because of them.”

Urban participants were more likely than rural participants to go to another community for support (OR = 1.57, 95% CI 1.03–2.37), though overall, participants sought support within their communities more often than outside (Table 3). Urban young women seeking support outside their community went to friends more often than relatives (62% vs. 38%). This may refer to them seeking support from friends in another neighbourhood, but within the same town. Rural young women seeking support outside their community went to relatives more often than friends (45% vs. 54%). This may reflect stronger family ties in rural areas. The poorest participants (OR = 0.49, 95% CI 0.27–0.88) and those that had not completed secondary education (OR = 0.44, 95% CI 0.29–0.66) were less likely to go to someone in a different community. When discussing these findings, some young women pointed out that they did not always have money to go to another community, and that people from other neighbourhoods did not respect them at times. On the other hand, another commented that some people in their own community look down on them and so that pushes them to look elsewhere.

The youngest participants (< 21) were less likely to go to people with children (OR = 0.45, 95% CI 0.31–0.66). The least educated (OR = 1.88, 95% CI 1.36–2.61), those that were cohabiting with a partner (OR = 1.74, 95% CI 1.18–2.56), and especially those with children (OR = 3.44, 95% CI 2.40–4.94) were more likely to go to others with children. In the discussion groups, young women who themselves had children explained that they sought out others with children because of shared experiences and empathy. One young woman said: “They won’t go around talking badly about you because they understand your situation.”

The younger participants (< 21 years) were more likely to go to others with limited education (OR = 1.86, 95% CI 1.21–1.85). Less educated women were more than four times as likely to go to others with limited education (OR = 4.23, 95% CI 2.98–6.02), and participants with children were more likely to turn to others with limited education (OR = 1.51, 95% CI 1.00–2.26). During discussion groups, some young women who had not completed secondary education explained they were embarrassed to ask for help from those who had been classmates but had successfully completed secondary school. One young woman explained that those who had not completed secondary school were more likely to have applied to government support programmes and so they were actually more knowledgeable about the process.

### Cluster adjustment

Treating each participant as a cluster in the ego-centric analysis, to allow for each young woman appearing repeatedly in the dataset, generally widened the confidence intervals as compared with non-clustered analysis. This did not change the interpretation of the findings. Taking into account clustering by community had very little effect on our results (see Additional file 2).

## Discussion

Marginalised young women sought support mostly from other women, usually living in the same community, often with children, especially if they had children themselves. We did not identify particular characteristics of young women associated with centrality (i.e. referral points that could be good disseminators of information). Rural areas were better connected than urban areas, but there were isolated participants in all areas. This suggests existing networks of peers may help in recruitment, particularly in rural areas, but peer recruitment is unlikely to reach all young women who might benefit from support programmes.

### Peer recruitment

Some of our findings are in line with homophily, the idea that people create bonds with people they consider similar to themselves in terms of factors such as race, sex, age, education [27, 28]. Previous research has reported female preference for female support. In school, many children associate by gender for play [29]. Controlling for family relationships, there is a preference for ties of the same gender [27], though, under stress, there appears to be a preference for female support from both men and women [30]. Participants in our study with children often went to others with children. Research in the United States showed women’s social networks decreased in size and intensity when they had children [31] and that mothers and grandmothers of adolescent mothers may be important sources of support [32]. Young women with limited education were more likely to go to others with limited education. Others have reported that students tend to self-organize into groups of similar educational achievement [33] and that similar educational achievement helped explain who women turned to for support related to issues of school and work [34]. About one half of the people young women went to were within 5 years of their own age and the poorest among them were less likely to seek support from someone of a similar age. This could reflect transactional and intergenerational sexual relationships [35], which may be more important for survival in the poorest young women [36]. Two-thirds of the people the young women in our study went to for support lived in the same community as themselves. Other authors have reported that individuals tend to associate with others that live nearby to themselves because of ease [37, 38]. Thus, our results show homophily related to gender, presence of children, education, and geography, but not age. In our study area, the population was homogenous in terms of religion and ethnicity; future studies wishing to use peers for recruitment should consider the possible impact of these factors. Two reviews have shown the effectiveness of peer-interventions for HIV prevention [39, 40]. Who exactly these peers should be for any given intervention is less clear. Our findings suggest reaching marginalised young women could be most effective through women who have similar lived experiences even if they are of different ages.

### Lessons for reaching marginalised young women

Network interventions refers to the practice of using networks to enact change. Valente reports on a number of different approaches that could be used to make changes to networks, whether to strengthen them to help spread information or to disrupt them to help prevent the spread of disease [41]. The first of these identifies central individuals who can influence change in others. In the second, segmentation, the population is split up into groups and then the goal is to change each group separately but simultaneously. The third, induction, aims to activate already existing networks ties. And the fourth, alteration, aims to change the network structure by adding or removing nodes and ties.

Our research was not able to identify characteristics of central young women that could spread information. However, we were able to identify characteristics of people in their broader networks that they turned to for support. They often went to peers (though not necessarily of the same age), and so peers could be important to affect change.

Peer recruitment is a method that has been used to reach marginalised populations. Reviews of recruitment strategies have identified respondent driven sampling, snowball sampling, collaboration with community organizations, social marketing, or a combination of these methods as methods for developing a list of potential participants or recruiting vulnerable populations [42, 43]. Respondent driven sampling, a method that builds on snowball sampling, aims to produce valid population estimates starting from a convenience sample [44, 45], though there are debates as to its accuracy [46]. Social marketing is the use of marketing strategies for social purposes [47]. Unlike much research that requires only a representative sample, interventions to improve health often seek to recruit as many eligible participants as possible.

Building on these strategies, we were interested to identify and to recruit an entire sub-population - all marginalised young women, without exception. Our recruitment strategy used peer recruitment along-side door-to-door recruitment and key community informants. Asking participants about multiple support networks may have prompted them to name more network members than they would have done otherwise. While our strategy was insufficient to reach the entire sub-population, our multi-pronged approach was efficient in quickly reaching most young women (90% of identified young women in 4–5 days/community). While the methods will need to be adapted to the local context, a multi-pronged approach appears beneficial.

Peer recruitment contributed most in rural areas. Other authors reported more dense connections in rural than urban areas [48] and that rural areas have more social capital (value inherent in social relationships) [49]. It was clear from doing field work in these communities that people in the rural communities were much more active in community activities, such as village meetings. In urban communities, fewer people were interested in this work, and many had taken advantage of better transportation to leave town for work or other reasons. The greater number of connections we found in rural communities suggests that spreading information through network leaders or inducing change by activating existing networks, as Valente suggests [41], may work more effectively in rural than in urban communities.

The relative homogeneity of these women’s networks may limit their access to opportunities. Homophilous ties among women in an organization were shown to be less beneficial to them than homophilous ties among men because more men were in high status positions [50]. Creating network connections with people outside their usual networks [51] is a promising route to providing participants with new opportunities. The INSTRUCT workshops introduce marginalised young women to programme officers who can help them to access programme resources and strengthens connections between young women. Altering the network, as Valente suggests [41], by building ties to government programme officers would allow information about available support programmes to enter the network via the programme officers, and diffuse amongst networks of young women. Not all young women are connected to each other, however, so we need multiple approaches to spread this information to all young women in each community.

Effective and inclusive recruitment is important to put the most marginalised young women in contact with government services, outside of the research context. Door-to-door recruitment is not feasible in routine practice for programme officers; however, in Nigeria, universal door-to-door visits by community health workers have been proposed to improve maternal and infant health outcomes [52]. In Botswana, health education assistants, who deliver basic health information and health education in communities and homes [53], could be a resource to share information on support programmes. Reaching all young women with home visits can be challenging especially for those that live in remote areas. Our findings suggest that young women are turning to people that are similar to themselves; recruitment through peers and key community members (possibly through existing community groups) may be quite effective, particularly in rural areas where participants were better connected.

### Strengths and limitations

Without contacting everyone in the target population, sociometric analysis can produce misleading results; it can identify participants as being isolated when they are not. We believe our multi-pronged recruitment strategy minimized these risks in an affordable way. Despite our multi-pronged approach, recruitment in urban areas was challenging. This could be due to the fact that there are fewer connections in urban areas or it could be that our recruitment strategies were less effective in urban areas. We tried to reduce the impact of incomplete populations of marginalised young women by using in-degree as our main measure of centrality, as it is relatively robust to missing data [54], and by using ego-centric analysis to describe participants’ overall support networks. Nonetheless, the challenges in recruiting in urban areas persisted; there is a need to explore new approaches to address this issue.

Most of the network literature is from work conducted in North America and Western Europe; few studies have explored social networks in Africa [55]. Our work contributes new knowledge and methodological insights to an area with limited research.

## Conclusion

Since marginalised young women seek support from others like themselves, outreach programmes should consider how to use or strengthen networks of women to identify and engage those who most need help from government structural support programmes. Methods that rely on social networks alone may be insufficient, and so a combination of approaches, including, for instance, peers, door-to-door recruitment, and key community informants, should be explored as a strategy for reaching marginalised young women for supportive interventions.

## Additional files

Additional file 1: Guiding questions for the discussion groups. This file includes the questions and findings that were used during the discussion groups with young women. (DOCX 15 kb)

Additional file 2: Clustering. This file explains how the authors accounted for repeated measures in the dataset using generalized linear mixed models. (DOCX 27 kb)

Additional file 3: Saturated and final models of young women’s support networks. This file shows the saturated and final models explaining who marginalised young women turn to for support. The saturated models show the impact of all the independent variables even if they were not significant in the final models. (DOCX 24 kb)

## Abbreviations

HIV: Human immunodeficiency virus
INSTRUCT: Inter-ministerial National Structural Intervention Trial
RDS: Respondent driven sampling
