# Supplementary material for: Can social network analysis help to include marginalised young women in structural support programmes in Botswana? A mixed methods study
Source: Int J Equity Health. 2019 Jan 18;18:12. doi: 10.1186/s12939-019-0911-8 (PMC6339404; doi:10.1186/s12939-019-0911-8)
Supplement: Supplementary file 1 — Guiding questions for the discussion groups. This file includes the questions and findings that were used during the discussion groups with young women. (DOCX 15 kb) [file 12939_2019_911_MOESM1_ESM.docx]

**Additional file 1 – Guiding questions for the discussion groups**

First, the facilitator described the survey participants.

- 3 out of every 4 participants were single
- 6 out of every 10 have a regular partner
- 6 out of every 10 have at least one child
- 2 out of every 3 have not completed secondary education
- 1 out of every 6 did not have enough food to eat in the last week (proxy for poverty)

Second, the facilitator described the people that young women from our survey said they went to for information.

- 9 out of every 10 support people were women
- 7 out of every 10 have children
- 6 out of every 10 are of a similar age
- Less than half have completed secondary education
- 2 out of every 3 are relatives (the others are mostly friends)
- 2 out of every 3 live in the same community
- 9 out of every 10 young women communicate face to face to get information
- 1 out of every 4 communicates by telephone
- 1 out of every 6 communicates using Facebook ©
- 1 out of every 14 communicates using WhatsApp ©

For each of the findings, the facilitator asked: “What do you think about this finding, based on your experience and what you know about your community?” Participants discussed each finding in turn, exploring whether they agreed with the findings or not and why. The facilitator then asked: “Why do you think young women choose people like themselves or other female family members to go to for information?”
