# Supplementary material for: Can social network analysis help to include marginalised young women in structural support programmes in Botswana? A mixed methods study
Source: Int J Equity Health. 2019 Jan 18;18:12. doi: 10.1186/s12939-019-0911-8 (PMC6339404; doi:10.1186/s12939-019-0911-8)
Supplement: Supplementary file 2 — Clustering. This file explains how the authors accounted for repeated measures in the dataset using generalized linear mixed models. (DOCX 27 kb) [file 12939_2019_911_MOESM2_ESM.docx]

**Additional file 2: Clustering**

Supplementary table 2 shows the effect of taking different types of clustering into account. Clustering (random effects) is used in this context to take account for the fact that both participants and support people could be repeated in this dataset. We also show a model where community was included as a cluster to account for repeated community level measures such as urban/rural status. We compare these to a model without any clustering (using the Mantel-Haenzsel method to calculate odds ratios). Clustered analysis is generally more conservative (i.e. wider confidence intervals). The table shows that there was little variation in the outcomes and in their interpretation.

**Supplementary table 2: Using generalized linear mixed models to account for repeated measures**

| **Cluster (random effect):** | **Participant** | | **Community** | | **Support person** | | **No cluster (Mantel- Haenszel)** | |
| --- | --- | --- | --- | --- | --- | --- | --- | --- |
|  | **OR** | **95% CI** | **OR** | **95% CI** | **OR** | **95% CI** | **OR** | **95% CI** |
| **Male support** | | | | | | | | |
| Urban | 0.64 | 0.46-0.91 | 0.65 | 0.48-0.87 | Model does not converge | | 0.63 | 0.47-0.85 |
| Poverty | 1.30 | 0.84-1.99 | 1.3 | 0.90-1.86 |  |  | 1.33 | 0.93-1.91 |
| Younger (<21) | 0.88 | 0.59-1.32 | 0.89 | 0.64-1.26 |  |  | 0.88 | 0.62-1.26 |
| Incomplete secondary | 0.78 | 0.56-1.09 | 0.78 | 0.59-1.04 |  |  | 0.74 | 0.55-0.99 |
| Cohabiting | 1.47 | 1.02-2.12 | 1.41 | 1.04-1.90 |  |  | 1.55 | 1.13-2.11 |
| Has children | 0.77 | 0.52-1.13 | 0.78 | 0.56-1.08 |  |  | 0.73 | 0.52-1.02 |
| **Similar Age (+- 5 years)** | | | | | | | | |
| Urban | 1.54 | 1.10-2.16 | 1.08 | 0.89-1.31 | 1.08 | 0.88-1.34 | 1.08 | 0.88-1.32 |
| Poverty | 1.08 | 0.82-1.41 | 0.67 | 0.52-0.86 | 0.65 | 0.49-0.86 | 0.67 | 0.52-0.87 |
| Younger (<21) | 0.64 | 0.45-0.91 | 1.22 | 0.97-1.55 | 1.24 | 0.96-1.61 | 1.23 | 0.97-1.56 |
| Incomplete secondary | 0.82 | 0.62-1.08 | 0.81 | 0.67-0.99 | 0.81 | 0.65-1.01 | 0.80 | 0.65-0.98 |
| Cohabiting | 0.81 | 0.60-1.09 | 0.84 | 0.67-1.04 | 0.82 | 0.65-1.05 | 0.82 | 0.66-1.02 |
| Has children | 1.02 | 0.74-1.39 | 1.02 | 0.82-1.28 | 1.02 | 0.80-1.31 | 0.99 | 0.79-1.25 |
| **Relative (vs. friend)** | | | | | | | | |
| Urban | 0.84 | 0.63-1.13 | 0.85 | 0.65-1.11 | 0.85 | 0.67-1.07 | 0.81 | 0.66-1.00 |
| Poverty | 1.46 | 0.98-2.17 | 1.38 | 1.06-1.79 | 1.44 | 1.05-1.98 | 1.32 | 1.02-1.71 |
| Younger (<21) | 0.89 | 0.62-1.27 | 0.91 | 0.72-1.15 | 0.90 | 0.68-1.20 | 0.91 | 0.71-1.16 |
| Incomplete secondary | 1.37 | 1.01-1.85 | 1.31 | 1.07-1.60 | 1.37 | 1.08-1.74 | 1.44 | 1.17-1.77 |
| Cohabiting | 1.20 | 0.85-1.68 | 1.16 | 0.93-1.45 | 1.18 | 0.90-1.54 | 1.17 | 0.93-1.46 |
| Has children | 1.07 | 0.76-1.52 | 1.11 | 0.88-1.39 | 1.12 | 0.85-1.47 | 1.08 | 0.86-1.36 |
| **Different community (vs same)** | | | | | | | | |
| Urban | 1.59 | 1.04-2.44 | 1.38 | 1.03-1.85 | 1.47 | 1.07-2.02 | 1.35 | 1.09-1.66 |
| Poverty | 0.47 | 0.26-0.85 | 0.54 | 0.40-0.72 | 0.44 | 0.28-0.69 | 0.60 | 0.45-0.79 |
| Younger (<21) | 1.16 | 0.70-1.95 | 1.03 | 0.80-1.32 | 0.99 | 0.68-1.45 | 1.05 | 0.82-1.35 |
| Incomplete secondary | 0.43 | 0.28-0.67 | 0.57 | 0.46-0.70 | 0.44 | 0.32-0.61 | 0.55 | 0.45-0.67 |
| Cohabiting | 0.63 | 0.39-1.04 | 0.67 | 0.52-0.86 | 0.59 | 0.40-0.85 | 0.69 | 0.54-0.89 |
| Has children | 1.25 | 0.76-2.06 | 1.14 | 0.90-1.46 | 1.16 | 0.80-1.67 | 1.15 | 0.90-1.46 |
| **Has children (vs. does not)** | | | | | | | | |
| Urban | 1.04 | 0.75-1.44 | 1.04 | 0.84-1.29 | 1.04 | 0.78-1.39 | 0.98 | 0.78-1.22 |
| Poverty | 1.15 | 0.73-1.80 | 1.14 | 0.84-1.54 | 1.20 | 0.81-1.79 | 1.19 | 0.88-1.61 |
| Younger (<21) | 0.46 | 0.31-0.68 | 0.52 | 0.40-0.66 | 0.45 | 0.32-0.63 | 0.52 | 0.41-0.67 |
| Incomplete secondary | 1.84 | 1.31-2.59 | 1.67 | 1.34-2.08 | 1.84 | 1.37-2.48 | 1.80 | 1.43-2.26 |
| Cohabiting | 1.75 | 1.19-2.59 | 1.63 | 1.25-2.11 | 1.81 | 1.28-2.57 | 1.72 | 1.32-2.25 |
| Has children | 3.43 | 2.36-4.98 | 2.88 | 2.27-3.65 | 3.76 | 2.67-5.27 | 2.74 | 2.15-3.49 |
| **Incomplete secondary** | | | | | | | | |
| Urban | 0.76 | 0.54-1.08 | 0.77 | 0.60-0.98 | 0.72 | 0.55-0.96 | 0.83 | 0.67-1.02 |
| Poverty | 1.27 | 0.80-2.03 | 1.17 | 0.90-1.54 | 1.22 | 0.84-1.78 | 1.14 | 0.88-1.48 |
| Younger (<21) | 1.81 | 1.18-2.77 | 1.68 | 1.31-2.15 | 1.94 | 1.37-2.75 | 1.62 | 1.25-2.09 |
| Incomplete secondary | 4.06 | 2.83-5.83 | 3.08 | 2.51-3.78 | 4.14 | 3.06-5.61 | 3.01 | 2.45-3.70 |
| Cohabiting | 1.04 | 0.70-1.54 | 1.00 | 0.80-1.26 | 1.01 | 0.73-1.39 | 1.00 | 0.79-1.27 |
| Has children | 1.44 | 0.95-2.16 | 1.42 | 1.12-1.81 | 1.56 | 1.12-2.17 | 1.44 | 1.12-1.84 |

Outcomes are characteristics of people that marginalized young women go to for support. Independent variables are characteristics of marginalized young women
