# Supplementary material for: Can social network analysis help to include marginalised young women in structural support programmes in Botswana? A mixed methods study
Source: Int J Equity Health. 2019 Jan 18;18:12. doi: 10.1186/s12939-019-0911-8 (PMC6339404; doi:10.1186/s12939-019-0911-8)
Supplement: Supplementary file 3 — Saturated and final models of young women’s support networks. This file shows the saturated and final models explaining who marginalised young women turn to for support. The saturated models show the impact of all the independent variables even if they were not significant in the final models. (DOCX 24 kb) [file 12939_2019_911_MOESM3_ESM.docx]

**Additional file 3 – Saturated and final models of young women’s support networks**

**Supplementary table 1: Generalized linear mixed models – Types of people that marginalized young women turn to for support**

This table describes the characteristics of our participants that were associated with being more or less likely to seek support from various types of people. The characteristics of the participants are the independent variables. The characteristics of the people they turn to for support are the outcomes (in bold). This analysis includes individual young women as clusters (random effect). The final model is displayed in Figure 3 of the main text.

|  | Saturated model | | Final model | | Saturated model | | Final model | |
| --- | --- | --- | --- | --- | --- | --- | --- | --- |
| Independent variables | OR | 95% CI | OR | 95% CI | OR | 95% CI | OR | 95% CI |
|  | **Male support** | | | | **Different community (vs same)** | | | |
| Urban | 0.64 | 0.46-0.91 | 0.69 | 0.49-0.97 | 1.59 | 1.04-2.44 | 1.57 | 1.03-2.37 |
| Poverty | 1.3 | 0.84-1.99 |  |  | 0.47 | 0.26-0.85 | 0.49 | 0.27-0.88 |
| Younger (<21) | 0.88 | 0.59-1.32 |  |  | 1.16 | 0.70-1.95 |  |  |
| Incomplete secondary | 0.78 | 0.56-1.09 |  |  | 0.43 | 0.28-0.67 | 0.44 | 0.29-0.66 |
| Cohabiting | 1.47 | 1.02-2.12 |  |  | 0.63 | 0.39-1.04 |  |  |
| Has children | 0.77 | 0.52-1.13 |  |  | 1.25 | 0.76-2.06 |  |  |
|  | **Similar Age (+- 5 years)** | | | | **Has children (vs. does not)** | | | |
| Urban | 1.54 | 1.10-2.16 |  |  | 1.04 | 0.75-1.44 |  |  |
| Poverty | 1.08 | 0.82-1.41 | 0.61 | 0.43-0.86 | 1.15 | 0.73-1.80 |  |  |
| Younger (<21) | 0.64 | 0.45-0.91 |  |  | 0.46 | 0.31-0.68 | 0.45 | 0.31-0.66 |
| Incomplete secondary | 0.82 | 0.62-1.08 |  |  | 1.84 | 1.31-2.59 | 1.88 | 1.36-2.61 |
| Cohabiting | 0.81 | 0.60-1.09 |  |  | 1.75 | 1.19-2.59 | 1.18 | 1.18-2.56 |
| Has children | 1.02 | 0.74-1.39 |  |  | 3.43 | 2.36-4.98 | 3.44 | 2.40-4.94 |
|  | **Relative (vs. friend)** | | | | **Incomplete secondary** | | | |
| Urban | 0.84 | 0.63-1.13 |  |  | 0.76 | 0.54-1.08 |  |  |
| Poverty | 1.46 | 0.98-2.17 |  |  | 1.27 | 0.80-2.03 |  |  |
| Younger (<21) | 0.89 | 0.62-1.27 |  |  | 1.81 | 1.18-2.77 | 1.86 | 1.21-2.85 |
| Incomplete secondary | 1.37 | 1.01-1.85 | 1.44 | 1.08-1.93 | 4.06 | 2.83-5.83 | 4.23 | 2.98-6.02 |
| Cohabiting | 1.2 | 0.85-1.68 |  |  | 1.04 | 0.70-1.54 |  |  |
| Has children | 1.07 | 0.76-1.52 |  |  | 1.44 | 0.95-2.16 | 1.51 | 1.00-2.26 |
